# Assessment of the pollution levels of potential toxic elements in urban vegetable gardens in southwest China

**DOI:** 10.1038/s41598-021-02069-6

**Published:** 2021-11-24

**Authors:** Jianing Gao, Dan Zhang, Ram Proshad, Ernest Uwiringiyimana, Zifa Wang

**Affiliations:** 1 Key Laboratory of Mountain Surface Processes and Ecological Regulation, Institute of Mountain Hazards and Environment, Chinese Academy of Sciences, #9, Block 4, Renminnanlu Road, Chengdu, 610041 Sichuan People’s Republic of China; 2 University of Chinese Academy of Sciences, Beijing, People’s Republic of China; 3 Institute of Atmospheric Physics, Chinese Academy of Sciences, Beijing, People’s Republic of China

**Keywords:** Environmental monitoring, Risk factors

## Abstract

Vegetable gardens are increasingly common in urban areas and can provide numerous societal benefits; however, contamination with potential toxic elements (PTEs) due to urbanization and industrialization is cause for concern. The present study aimed to assess the source of contamination and pollution levels in urban garden soils, as well as the health risks for adults and children consuming vegetables grown in such environments. Various types of vegetable samples and their corresponding soils from 26 community gardens were collected throughout Chengdu City in southwestern China. The results showed that leafy vegetables, particularly lettuce leaves and Chinese cabbage, had relatively higher levels of Cd (0.04 mg/kg FW) and Pb (0.05 mg/kg FW), while higher levels of As (0.07 mg/kg FW), Cr (0.07 mg/kg FW), and Hg (0.003 mg/kg FW) were found in amaranths, tomatoes, and Houttuynia cordatas, respectively. The pollution indices revealed that the vegetable purplish soils were relatively more polluted by Cd and As, and the concentrations of these metals in vegetables were correlated with their concentrations in the soils. Principal component analysis grouped the PTEs in two dimensions that cumulatively explained 62.3% of their variation, and hierarchical clustering identified two distinct clusters indicating that Cr originated from a unique source. The health risk assessment revealed that exposure to As and Cd induced the greatest non-carcinogenic risk, whereas Cr was most likely to cause cancer risks. Furthermore, contaminated vegetable consumption was riskier for children than adults. The critical factors contributing to PTE contamination in vegetable gardens were determined to be vegetable species, total soil element content, soil pH, and soil organic matter content. Overall, Cr and As pollution present the greatest concern, and community health care services must enact more effective regulatory and preventative measures for urban gardens in terms of PTEs.

## Introduction

Soil is considered a vital component contributing to human survival on the earth because of its role as a nutrient benefactor for plant growth; however, soil is also anticipated to be a principal recipient of potential toxic elements (PTEs)^1–3^. The presence of PTEs in soil has emerged as a significant problem in many parts of the world, including China, as a result of abrupt urbanization and industrialization in combination with rapid population growth over the last few years^4,5^. Pollution from PTEs affects the soil composition, texture, and function of urban garden areas, as well as introverted plant root development, thereby reducing crop production^6,7^. The abundance, persistence, and environmental toxicity of PTEs can directly or indirectly induce detrimental consequences on human health by propagating through the food chain^6,8,9^. Therefore, the scientific research community has recently dedicated more attention to investigating potential means of particulate deposition and monitoring PTE levels in the environment^6,10,11^.

In China, pollution with toxic elements has become an increasingly serious issue, leading to increased public concern about the possible accumulation of heavy metals in agricultural soils, which ultimately poses a major threat to human health through the food chain^3,12^. Vegetable consumption is a key part of Chinese people’s diets, and it therefore represents a likely pathway by which toxic element contamination can climb the food chain and affect human health^5,8,13^.

Urban vegetables garden is one of major form of urban agriculture in the world as well as China that act as an important feature of urban food security and sustainable urban food systems^14,15^. Urban gardens are characterized by a wide range of agricultural and industrial activities and are found in the urban areas^16^. Because urban regions are often subjected to several emission sources of vegetable garden soils contamination by heavy metals is more severe and complicated. Urban garden soils and vegetables contamination can be burning issues in the subject of ecological risks and human health hazards. Industrial operations, irrigation with polluted water and atmospheric deposition in the urban regions resulting metal pollution in soil and vegetables^17–19^. Soil acts as reservoir of toxic metals accumulation that transfer to vegetables and enter into human food chain that cause non-carcinogenic and carcinogenic health hazards due to consumption of contaminated vegetables^17,20^.

Southwest China is the most prominent commercial and industrial region in the country, so urban soil in this region is significantly influenced by the emission of pollutants from various surrounding industries and heavy traffic. Numerous studies have investigated the PTE pollution in agricultural soils in southeastern Chinese provinces, such as Zhejiang^21,22^, Jiangsu^23,24^, Guangdong^25^, and Shandong^24^. However, to our knowledge, no comprehensive assessments have focused on PTE pollution in urban garden soils and vegetables in southwestern China. Moreover, there is generally insufficient information regarding PTE pollution in this region. Therefore, present study was conducted to measure the PTE levels in urban garden soils and vegetables and to determine their possible sources of metals with possible health risks for adults and children exposed to PTEs in urban vegetable gardens. The PTE concentrations in urban soil and vegetables were compared with the results of several studies conducted around the world to assess the relative pollution levels of southwest China’s urban vegetable gardens in a global context.

## Materials and methods

### Study area

This study was conducted in Chengdu City, Sichuan Province (102°54′E–104°53′E, 30°05′N–31°26′N), which is one of the most cardinal megacities in China and in the world, with a population of more than 16 million, where the urban population density is approximately 6500 people/km^2^. Topographically, Chengdu City is located in the western part of the Sichuan Basin, surrounded by the Longquan Mountains in the east and the Qionglai Mountains in the west^26^. It has a subtropical monsoon humid climate with a mean annual temperature of 15.5 ℃ and a mean annual rainfall of 1000 mm. The main soil types present in this region are lateritic red soil, fluvo soil, purplish soil, and paddy soil.

### Sampling

**Figure 1:**
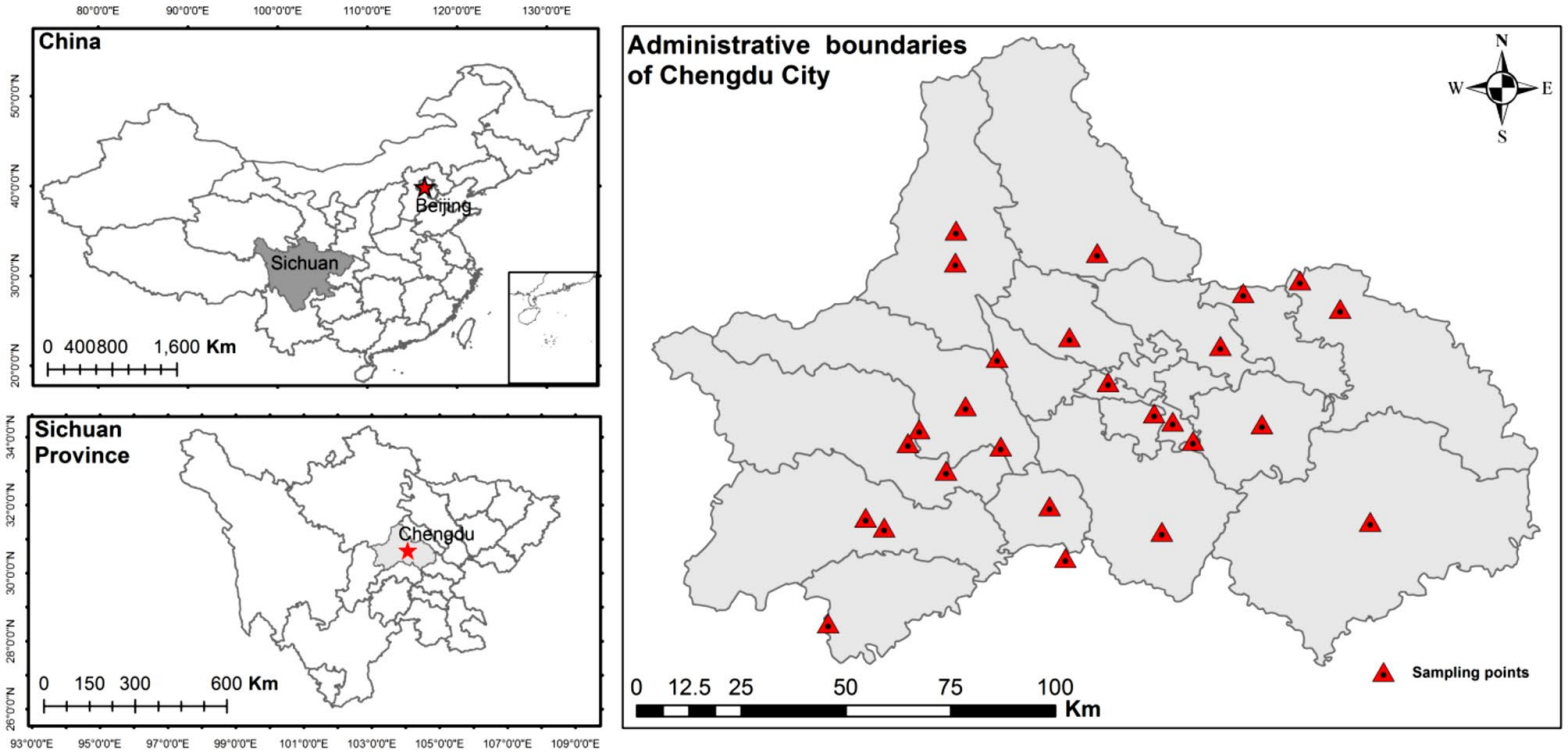
Map of the study area showing the sampling sites in Chengdu City, Southwest China. The maps were created using ArcMap version 10.4 software (https://desktop.arcgis.com/). The administrative boundaries data were accessed via http://211.159.153.75/.

A total of 113 topsoil (from 0 to 20 cm deep) samples, including 63 purplish soils and 50 yellow soils, and their corresponding vegetable samples (0.5 kg edible part of each) were collected from 26 community gardens in the sampling area, as shown in Fig. 1. Up to four samples were collected from different locations in each garden, and analyzed individually, depending on the field survey characteristics (i.e., soil type, vegetable species, and regional specifications). The vegetables analyzed included 51 leafy vegetables (i.e., 15 amaranths, 21 Chinese cabbage, 15 lettuce leaves), 24 rootstock vegetables (i.e., 12 carrots and 12 Houttuynia cordatas), 26 legume vegetables (i.e., 12 pea pods and 14 cowpeas), and 12 solanaceous vegetables (12 tomatoes). All soil and vegetable specimens were blended from three random subsamples.

### Pre-treatment and analysis of soil and vegetable samples

All soil samples were homogenized, air-dried, pulverized, and passed through a 2-mm sieve. The soil pH was measured using a pH meter with a soil:water ratio of 1:2.5^27^. The soil organic matter (SOM) was determined via wet oxidation at 180 °C with a mixture of potassium dichromate and sulfuric acid^28^. The total Cd, Pb, Cr, As, and Hg were dissolved in pure hydrochloric acid and nitric acid in the microwave.

Vegetables were carefully rinsed with distilled water to remove dust and impurities attached to the leaf and/or root surface. All edible parts were extracted with nitric acid, hydrogen peroxide, and hydrofluoric acid to determine the concentrations of PTEs. The concentrations of Cd, Pb, Cr, and As in soils and plants were measured in triplicate using inductively coupled plasma mass spectrometry (ICP-MS; Agilent 7700x, USA). The concentration of Hg was measured with an atomic fluorescence spectrometer (AFS; PF6-3, China). Quality assurance and quality control were performed by duplicate samples, blanks and standard substances. The relative standard deviation (RSD) of the duplicates was less than 6%, and the recovery rates of the standard substances were 104% and 95%, respectively. All PTE concentrations were determined on a dry weight (DW) basis, and are expressed herein in units of mg/kg DW.

### Ecological risk assessments

#### Geo-accumulation index

The geo-accumulation index (*I*_geo_) was determined to assess the contamination levels of PTEs in the study area^29^. The *I*_geo_ values were calculated using Eq. (1),

1 $$ \mathop I\nolimits_{geo} = \mathop {\log }\nolimits_{2} \left( {\frac{{\mathop C\nolimits_{k} }}{{1.5\mathop B\nolimits_{k} }}} \right) $$

where *C*_*k*_ (mg/kg) is the measured PTE concentration in the soil and *B*_*k*_ (mg/kg) is the geochemical background value of PTEs in the studied soil. The constant 1.5 was employed to minimize the probable variations in the background values^30^. The *I*_*geo*_ for contamination levels were classified as follows: Class I (*I*_*geo*_ ≤ 0); Class II (0 < *I*_*geo*_ ≤ 1); Class III (1 < *I*_*geo*_ ≤ 2); Class IV (2 < *I*_*geo*_ ≤ 3); Class V (3 < *I*_*geo*_ ≤ 4); Class VI (4 < *I*_*geo*_ ≤ 5); Class VII (*I*_*geo*_ > 5)^31^.

#### Pollution index

The pollution index (*PI*) is the ratio between the total detected PTE contents in soil and their corresponding background levels^32^. This parameter was calculated using Eq. (2),

2 $$ PI = \frac{{\mathop C\nolimits_{k} }}{{\mathop B\nolimits_{k} }} $$

The degree of pollution can be classified as follows: low level (*PI* < 1); moderate level (1 ≤ *PI* < 3); high level (*PI* ≥ 3)^33^.

#### Pollution load index

The pollution load index (PLI) is an integrated pollution index, which is determined as the *n*^th^ root of the pollution index, as described by Eq. (3),

3 $$ PLI = \left( {\mathop {PI}\nolimits_{k1} \times \mathop {PI}\nolimits_{k2} \times \cdots \times \mathop {PI}\nolimits_{kn} } \right)^{{{\raise0.7ex\hbox{$1$} \!\mathord{\left/ {\vphantom {1 n}}\right.\kern-\nulldelimiterspace} \!\lower0.7ex\hbox{$n$}}}} $$

where *PI*_k1_, *PI*_k2_, and *PI*_kn_ are the *PI* values of element *k*1, *k*2,…*k*n, and *n* is the number of measured elements. A value of *PLI* > 1 indicates that the studied soil was polluted by PTEs^33^.

### Bio-accumulation and health risk assessment

#### Bio-accumulation factor

The bio-accumulation factor (BAF) is an index of the ability of the edible part of a vegetable to accumulate a particular metal ion with respect to its concentration in the soil substrate. This factor can be calculated using Eq. (4),

4 $$ BAF = \frac{{\mathop C\nolimits_{vegs} }}{{\mathop C\nolimits_{soil} }} $$

where *C*_vegs_ (mg/kg DW) and *C*_soil_ represent the PTE concentrations in the edible part of vegetables and in the soil, respectively.

#### Health risk assessment

We have determined metals concentration in dry weight basis but for daily intake estimation, we need to convert metal concentration in fresh weight basis^34^ using Eq. (5),

5 $${F}_{wc}={C}_{dw}\left[\frac{100-W}{100}\right]$$

where, ${F}_{wc}$ is the fresh weight concentration; ${C}_{dw}$ indicates dry-weight concentration and W represents percent water content in individual vegetables.

The estimated daily intake (*EDI*) can be determined using Eq. (6),

6 $$ \mathop {EDI}\nolimits_{vegs} = \frac{{\mathop C\nolimits_{vegs} \times \mathop {DCR}\nolimits_{vegs} \times EF \times ED}}{BW \times AT} $$

where $\mathop C\nolimits_{vegs}$ indicates the metal concentration in vegetables in fresh weight basis (Wang et al., 2020); *DCR*_vegs_ represents the daily average consumption rate of vegetables in this area (76 g/day for adults and 59 g/day for children)^35^; EF is the exposure frequency (350 days/year)^36^; ED is the exposure duration (76.3 years for adults and 6 years for children)^35,36^; BW is the average body weight (60 kg for adults and 25 kg for children)^37^; AT is the average time for non-carcinogens (365 × ED)^38^.

The risk assessment evaluated non-carcinogenic and carcinogenic risks associated with PTEs via diet exposure pathways. The non-carcinogenic risk of individual toxic elements, expressed as the hazard quotient (*HQ*), was calculated using Eq. (7),

7 $$ HQ = \frac{{\mathop {EDI}\nolimits_{k} }}{{\mathop {RFD}\nolimits_{k} }} $$

where *RFD*_k_ is the oral reference dose of the PTE. The RFD values for Cd, Pb, Cr, As, and Hg are 0.0005, 0.0035, 0.003, 0.0003, and 0.0001 mg/kg/day, respectively^38,39^.

The non-carcinogenic risk of multiple elements, expressed as the hazard index (*HI*), was determined from Eq. (8),

8 $$ HI = \sum {\mathop {HQ}\nolimits_{kn} } $$

where a value of *HI* > 1 indicates that there is an increasing non-carcinogenic risk, and vice versa for *HI* < 1.

The carcinogenic risk (*CR*) via oral exposure pathways can be evaluated from Eq. (9),

9 $$ CR = \mathop {EDI}\nolimits_{k} \times \mathop {SF}\nolimits_{k} $$

where *SF*_*k*_ is the oral slope factor for different PTEs. The *SF* values for Cd, Pb, Cr, and As are 5 × 10^–5^, 8.5 × 10^–3^, 41, and 1.5 mg/kg/day, respectively^36,39^. The acceptable cancer risk range is between 10^–6^ and 10^–4^. Values surpassing 10^–4^ are considered to pose significant health effects and risks while below 10^–6^ are viewed as lower health effect ^36^.

The cumulative carcinogenic risk (*TCR*) can be computed from Eq. (10),

10 $$ TCR = \sum {\mathop {CR}\nolimits_{kn} } $$

### Data analysis

Basic statistical analysis of the raw data was carried out using SPSS 20.0 software. One-way analysis of variance (ANOVA) was performed to determine the significant differences in PTE concentrations in vegetable and soil samples. Comparisons of means were carried out via the Duncan test at a 5% level of significance. Spearman correlations, principal component analysis (PCA), and cluster analysis (CA) based on squared Euclidean distances were applied to evaluate possible sources of the PTEs in the study area. The maps were created using ArcGIS version 10.4 software.

### Ethics approval and consent to participate

All the experimental protocols involving plants adhered to relevant ethical guidelines.

Vegetables collection permission from community gardens was obtained verbally from their owners.

## Results

### PTE concentrations in garden soils

The PTE concentrations and physicochemical properties of the studied garden soils are presented in Table 1. The data were not normally distributed (Kolmogorov–Smirnov test, *p* < 0.05), except in the case of SOM and Hg. The pH of the 113 soil samples ranged from 4.29 to 8.05 with an average value of 6.55, which suggested that the soil in study area was mostly neutral. The SOM content ranged from 10.25 to 153.88 g/kg, revealing that the SOM of garden vegetable soils in Chengdu City varied greatly. The concentrations of Cd, Pb, Cr, As, and Hg in the soil were 0.10–0.69, 11.59–82.60, 59.21–143.14, 8.17–61.57, and 0.01–0.10 mg/kg, with average concentrations of 0.22, 35.29, 90.91, 29.57, and 0.06 mg/kg, respectively (Table 1). According to the background values of PTEs in the superficial soil of Sichuan Province^40^, most of samples exceeded the background values for all elements. The topsoil samples were significantly enriched by Cd, followed by As (96%), Cr (77.9%), Hg (52%), and Pb (51.3%). There are only a few soil samples wherein the Pb, Cr, and Hg concentrations exceeded the regulation values, except in the case of Cd (16.8%) and As (19.5%).

**Table 1:** Characteristics of garden vegetable soils in Chengdu City.

Properties	Concentrations (*n* = 113)				SD	CV	BV	RV	p(K-S)
	Min	Max	Mean	Median					
pH	4.29	8.05	6.55	6.69	0.87	0.13			0.038
SOM (g/kg)	10.25	153.88	55.63	55.69	26.99	0.49			0.521
Cd (mg/kg)	0.10	0.69	0.22	0.18	0.13	0.57	0.08	0.3	0.000
Pb (mg/kg)	11.59	82.60	35.29	31.01	14.33	0.41	30.9	90	0.004
Cr (mg/kg)	59.21	143.14	90.91	86.30	19.55	0.21	79	150	0.033
As (mg/kg)	8.17	61.57	29.57	27.51	13.12	0.44	10.4	40	0.015
Hg (mg/kg)	0.01	0.10	0.06	0.06	0.02	0.34	0.061	1.8	0.914

*SD* standard deviation, *CV* coefficient of variation, *BV*  background values (CNEMC, 1990), *RV* regulation values (CEPA, 2018), *p(K-S)*  *p* value of Kolmogorov–Smirnov test.

Spearman correlation analysis, PCA, and CA were used to determine the possible sources of soil PTEs in the study area. Table 2 presents the correlation coefficients between soil properties and PTEs in the garden vegetable soils. Significant negative correlations were observed between pH and Cr (*r* = –0.260, *p* < 0.01), pH and As (*r* = –0.271, *p* < 0.05), SOM and As (*r* = –0.279, *p* < 0.01), and Hg and As (*r* = –0.249, *p* < 0.01). A significant positive correlation at the level of 0.05 or 0.01 was observed between the elemental pairs Cd–Pb (0.416), Cd-Cr (0.293), Pb-Cr (0.259), Pb-As (0.270), Cr-As (0.278), and Pb-SOM (0.231); the similar ranges of these correlations indicated that the elements possibly originated from the same source^41^.

**Table 2:** Spearman correlation matrix for soil properties and PTEs in the vegetable soils of Chengdu City (*n* = 113).

	pH	SOM	Cd	Pb	Cr	As	Hg
pH	1						
SOM	0.133	1					
Cd	0.005	0.034	1				
Pb	−0.103	0.231*	0.416**	1			
Cr	−0.260**	0.183	0.293**	0.259**	1		
As	−0.271**	−0.279**	0.175	0.270**	0.278**	1	
Hg	0.128	0.126	−0.095	0.109	−0.179	−0.249**	1

*Significant correlation at 0.05 level.

**Significant correlation at 0.01 level.

The results of PCA obtained by applying varimax rotation with Kaiser Normalization for PTE concentrations in garden vegetable soils are shown in Table S1. The eigenvalue of the first extracted factor was much greater than 1, and the second also exceeded one after the matrix rotation. These results indicated that PCA reduces the initial dimensions of the dataset to two components, which cumulatively explain 62.3% of the data variation. The first principal component (PC1) explains 36.83% of the total variance and loads heavily on Cd, Pb, and Cr, while As was only partially accounted for. The second principal component (PC2), dominated by Hg, accounts for 25.47% of the total variance. The CA results for PTEs in garden soils are presented as a dendrogram in Fig. S1, which displays two clusters: (1) Cd-Hg-Pb-As; (2) Cr. These CA results suggested at least two different sources of PTEs in the garden vegetable soils. Based on the statistical analysis results, we can consider that Cd, Pb, and Hg exclusively (and As partially) had a common source, while Cr had a distinct source.

### Pollution indices

**Figure 2:**
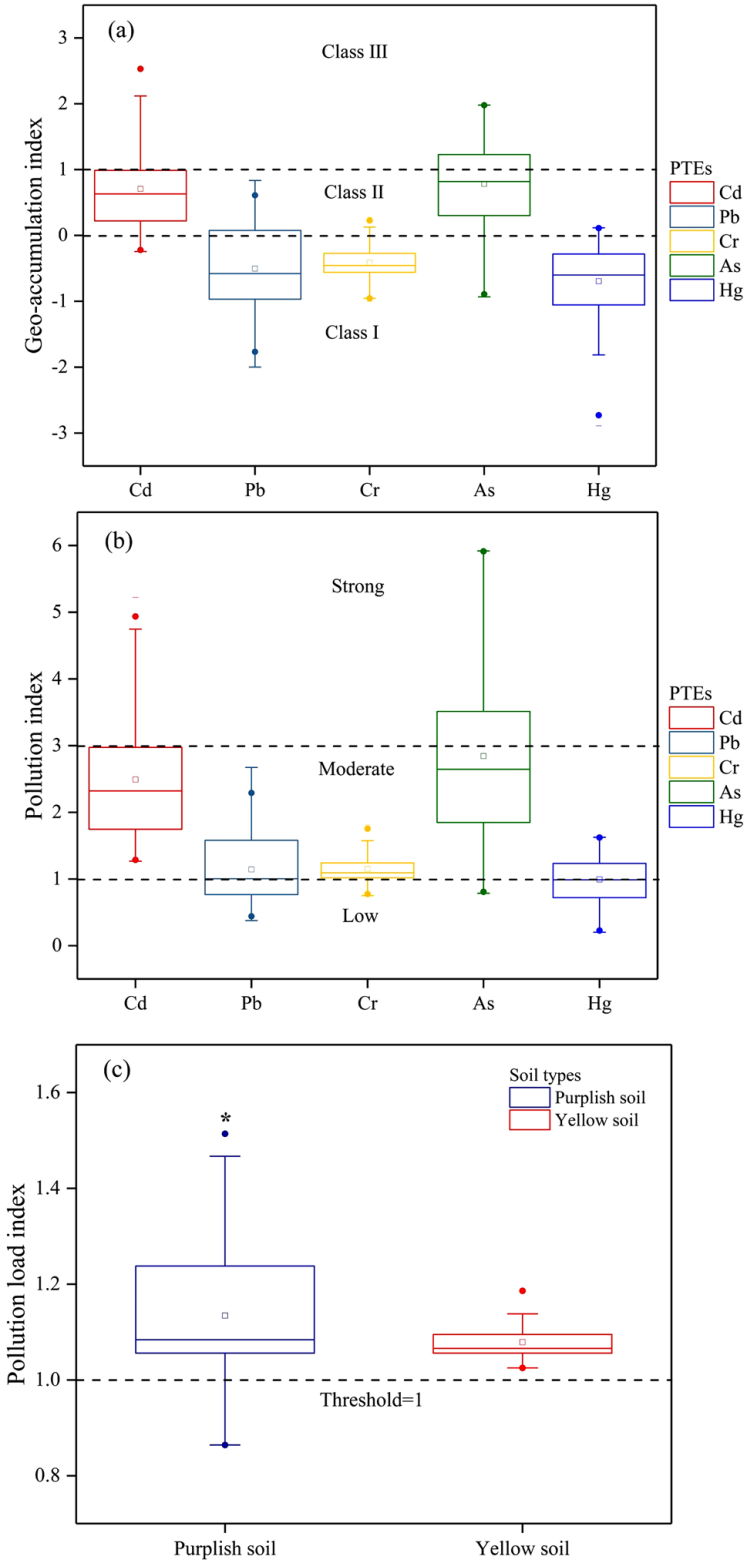
Pollution indices of PTEs in soils; **(a)** characteristics of geo-accumulation indices of PTEs in soils (Class I = uncontaminated; Class II = uncontaminated to moderately contaminated; Class III = moderately contaminated); **(b)** characteristics of pollution indices (low, moderate, and strong represent the contamination degrees); **(c)** characteristics of pollution load indices depending on soil type (small boxes inside the box plots designate the mean values, and (*) represents the significant differences of the PLI values based on the Duncan test, *p* < 0.01).

The *I*_geo_ and *PI* values were considered jointly as indices to assess the contamination levels of PTEs in the soil samples^41,42^, and the results based on pollution indices are shown in Fig. 2.

The *I*_geo_ values of Cd ranged from –0.24 to 2.53, and 57.5% and 24.8% of sampling sites belonged to Class II and Class III, respectively. For As, 59.3% and 31.9% of the samples belonged to Class II and Class III, respectively. These results are consistent with uncontaminated to moderate contamination of Cd and As in most sampling sites, with a high proportion of moderate contamination conditions. The *I*_geo_ values of Cr and Hg ranged from –1.0 to 0.27 and –2.89 to 0.12, respectively, and 11.5% and 8% of the respective samples belonged to Class II. These results suggested that most sampling sites were not contaminated with Cr or Hg, although a few samples were contaminated with these PTEs. Compared with Cr and Hg, Pb exhibited relatively higher *I*_geo_ values in Class II, accounting for 27.4% (Fig. 2a). Accordingly, the *PI* results revealed that the contamination level of Cd and As for the majority of the sampling sites were at a moderate level. Additionally, the *PI* values for Cr, Hg, and Pb indicated relatively low contamination of these elements in most of the sampling sites (Fig. 2b).

The *PLI* values were calculated as a comprehensive pollution index, and these results are also presented in Fig. 2. The *PLI* values of purplish and yellow soil were in the range of 0.86–1.51 and 1.03–1.19, with average values of 1.12 and 1.08, respectively, and the purple soils were significantly more polluted compared with yellow soils (*F* = 8.12, *p* < 0.01).

### PTE concentrations in vegetables

**Figure 3:**
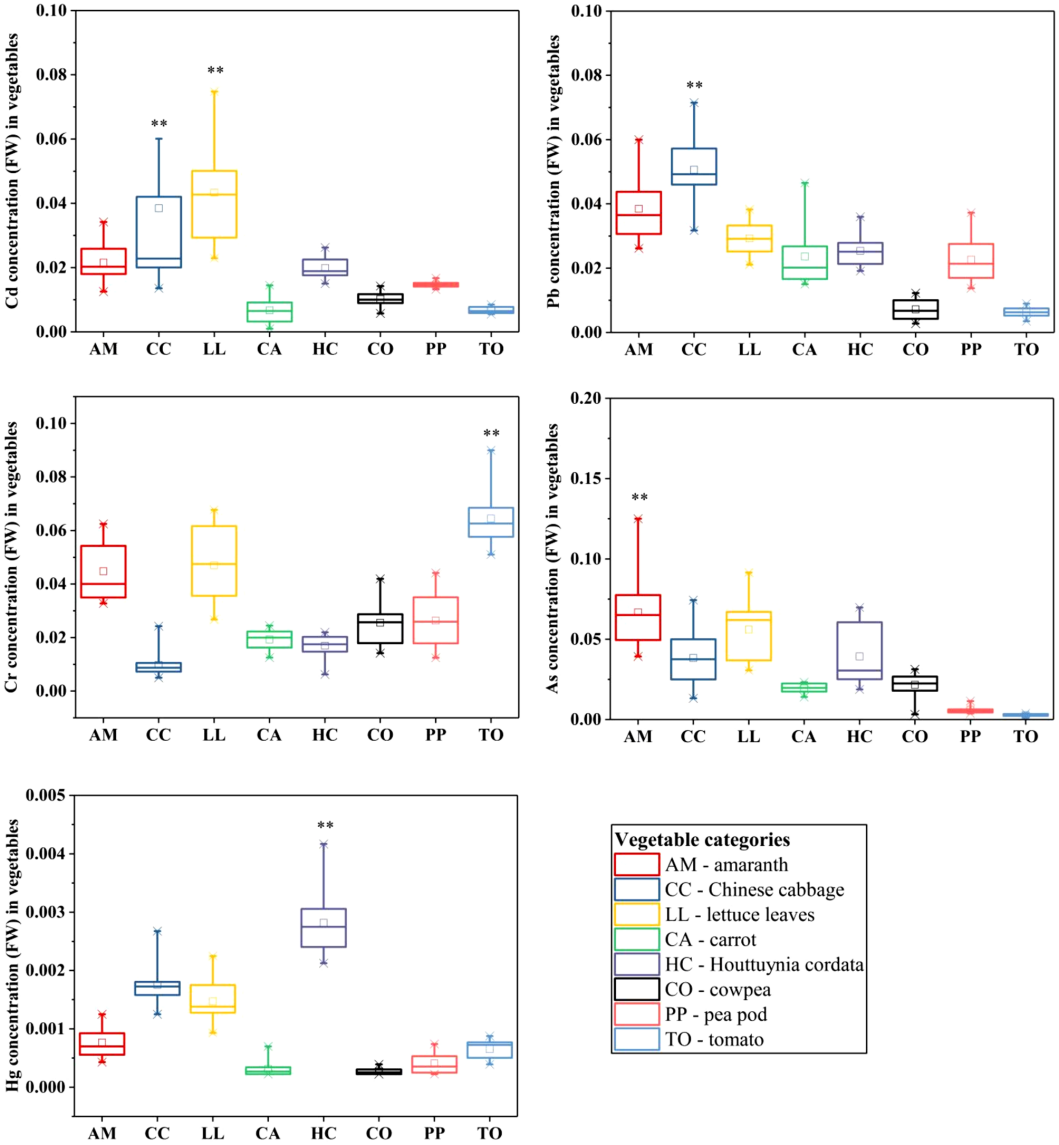
Concentration of PTEs in vegetables (FW) (little box in the middle of the box plots represent the mean values, and (**) represent the significant differences of the mean values for PTEs based on the Duncan test, *p* < 0.01).

The concentrations (on fresh weight basis, FW) of PTEs in the edible part of vegetable harvested from the study area are presented in Fig. 3. The concentration of Cd in the Chinese cabbage and lettuce leaves were in the range of 0.01–0.24 and 0.02–0. 08 mg/kg FW, with mean values of 0.04 and 0.04 mg/kg FW, respectively, which were significantly higher than those in other vegetables (*F* = 6.51, *p* < 0.001). The highest average concentrations of As and Pb were in the amaranths and Chinese cabbage (0.07 and 0.05 mg/kg FW, respectively); however, the highest average concentrations of Cr and Hg were in the tomatoes and Houttuynia cordatas (0.07 and 0.003 mg/kg FW, respectively). There were few vegetable samples in this study area whose PTEs concentrations exceeded the threshold values^43^.

The PTE concentrations in the four vegetable categories are summarized in Table S2. The average concentration of Cd decreased in the order, leafy (0.04 mg/kg FW) > rootstock (0.01 mg/kg FW) ≥ legume (0.01 mg/kg FW) ≥ solanaceous (0.01 mg/kg FW); Pb concentration decreased in the order, leafy (0.04 mg/kg FW) > rootstock (0.03 mg/kg FW) > legume (0.01 mg/kg FW) ≥ solanaceous (0.01 mg/kg FW); Cr decreased in the order, solanaceous (0.07 mg/kg FW) > leafy (0.03 mg/kg FW) ≥ legume (0.03 mg/kg FW) > rootstock (0.02 mg/kg FW); As decreased in the order, leafy (0.05 mg/kg FW) > rootstock (0.03 mg/kg FW) > legume (0.01 mg/kg FW) > solanaceous (0.003 mg/kg FW); and Hg decreased in the order, rootstock (0.002 mg/kg FW) > leafy (0.001 mg/kg FW) ≥ solanaceous (0.001 mg/kg FW) > legume ( less than 0.001 mg/kg FW ). These results indicated that leafy and rootstock vegetables were prone to pollution by Cd, Pb, As and Hg, whereas solanaceous (tomatoes) plants were vulnerable to pollution by Cr.

### Non-carcinogenic and carcinogenic risks for local adults and children

The total EDIs of PTEs through a vegetable diet for adults and children in the study area are presented in Table S3. The highest average EDI values for Cd were 0.053 and 0.098 mg/kg/day in lettuce leaves for adults and children, respectively. Similarly, the highest Pb values were 0.061 and 0.115 mg/kg/day in Chinese cabbage; the highest Cr values were 0.078 and 0.146 mg/kg/day in tomatoes; the highest As values were 0.081 and 0.151 mg/kg/day in amaranths; and the highest Hg values were 0.0034 and 0.0064 mg/kg/day in Houttuynia cordatas. The aforementioned EDIs of PTEs were significantly different among the studied vegetables, and those determined for children were statistically greater than the values for adults.

**Figure 4:**
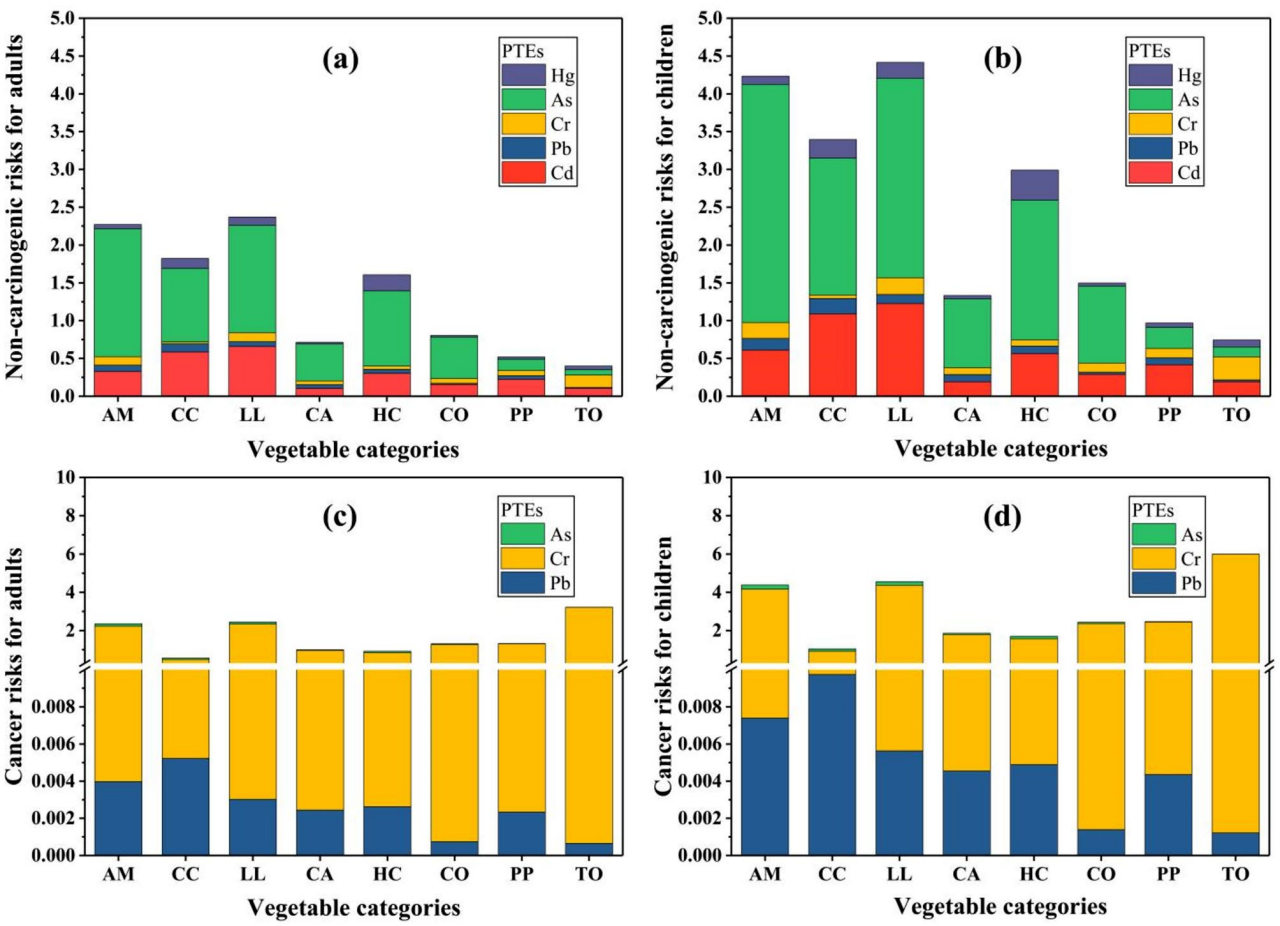
Health risks for adults and children from different vegetables (*AM* amaranths, *CC* Chinese cabbage, *LL* lettuce leaves, *CA* carrots, *HC* Houttuynia cordatas, *CO*  cowpeas, *PP* pea pods, *TO*  tomatoes).

The non-carcinogenic risks (HI) of PTEs for local adults and children are described in Fig. 4a,b. For adults, the HI values of lettuce leaves, amaranths, Chinese cabbage, and Houttuynia cordatas (2.37, 2.27, 1.82, and 1.61, respectively) were higher than the threshold value one (Fig. 4a). For children, the HI values of lettuce leaves, amaranths, Chinese cabbage, Houttuynia cordatas, cowpeas and carrots (4.42, 4.23, 3.40, 2.99, 1.50 and 1.33, respectively) were 1.86 times higher than the analogous values in adults (Fig. 4b). The average contribution of PTEs for adults and children followed the order, As (54.05%) > Cd (24.39%) > Cr (10.32%) > Hg (6.52%) > Pb (4.72%). Therefore, As poses the highest risks for local adults and children, followed by Cd and Cr.

The cancer risks from PTEs for local people are also shown in Fig. 4c,d. Relative to Cd, the toxic elements Pb, Cr and As were associated with higher cancer risks, particularly for Cr and As. The total combined risk for adults decreased in the following order: tomatoes (3.22) > lettuce leaves (2.45) > amaranths (2.35) > pea pods (1.32) > cowpeas (1.31) > carrots (1.00) > Houttuynia cordatas (0.92) > Chinese cabbage (0.56) > 10^–4^ (Fig. 4c). The total risk for children decreased in the order: tomatoes (6.00) > lettuce leaves (4.56) > amaranths (4.39) > pea pods (2.47) > cowpeas (2.44) > carrots (1.86) > Houttuynia cordatas (1.71) > Chinese cabbage (1.05) > 10^–4^, indicating that children consume vegetables at higher risk than adults (Fig. 4d).

## Discussion

### PTE contamination levels in Chengdu urban vegetable gardens relative to other parts of the world

To compare the PTE concentrations in Chengdu urban vegetable soil with studies conducted in other areas, the PTE concentrations determined in vegetable soils from other regions are compiled in Table 3. The mean concentrations of Cd, Pb, and Hg from Chengdu City were lower than the corresponding mean values from Hangzhou, Wuwei, Xiangyang, Tongling, Ghent, Melbourne, Christchurch, Marseille, Grand Nancy, and Nantes, but higher than the mean values from Marrakech. The city of Marrakech is located in the southwestern region of Morocco, and the area is characterized by soils with high pH and SOM^44^. The elevated pH and/or the formation of metal–organic compounds in the soils there may reduce the mobility of some PTEs, such as Cd and Pb^6,8^. In contrast, acidic conditions tend to promote the movement of toxic metals as a result of proton competition and decreased availability of negatively-charged binding sites^45^. In addition, such conditions increase ionic metal speciation because of the weak binding effect of SOM on soil components, which may further enhance the mobility and bioavailability of metals^46^. This could be a reason for the high Cd, Pb, and Hg levels detected in the soil from Hangzhou, Wuwei, Xiangyang, Tongling, Ghent, Marseille, Grand Nancy, and Nantes. Meanwhile, the average concentrations of Cr and As in soils from Chengdu City were consistently greater than the values in all other studies, and the concentrations varied highly among the different locations (Table 3). This indicates that some of the PTEs in urban vegetable soils have higher spatial variability due to the different soil types, soil properties, climates, levels of development, and human activities in the different regions^5,47–49^.

**Table 3:** Descriptive statistics of PTEs in urban and/or suburban garden vegetable soils (values in brackets are the mean values).

Study area	Sample number	Concentration (range, mean, mg/kg)					Soil property (range, mean)		
		Cd	Pb	Cr	As	Hg	pH	SOM (g/kg)	Reference
Chengdu (China)	113	0.10–0.69 (0.22)	11.59–82.60 (35.29)	59.21–143.14 (90.91)	8.17–61.57 (29.57)	0.01–0.10 (0.06)	4.3–8.0 (6.6)	10.3–153.9 (55.6)	This study
Hangzhou (China)	268	0.16–2.14 (0.72)	11.52–191.27 (68.64)	26.78–68.99 (47.74)	5.22–52.14 (15.51)	0.12–1.45 (0.76)	4.5–8.4 (6.4)	6.9–59.7 (23.9)	Liu et al., 2013
Wuwei (China)	97	0.10–1.53 (0.42)	15.33–27.89 (20.76)	40.08–60.48 (53.12)			7.4–8.3 (7.9)	10.4–52.3 (29.7)	Bai et al., 2014
Xiangyang (China)	136		24.25–132.55 (39.44)	31.3–70.3 (55.36)	3.48–11.89 (8.35)	0.01–0.54 (0.13)	7.0–8.3 (7.6)	35.3–80.0 (47.6)	Wang et al., 2017
Tongling (China)	44	0.2–3.6 (1.2)	19–198 (66)				5.7–7.9 (7.1)	13.3–90.5 (25.2)	Xu et al., 2013
Ghent (Belgium)	26	0.16–4.91 (0.53)	14.86–303.3 (97.5)	8.69–143.6 (26.4)	1.97–8.52 (4.39)		(5.9)	36–264	Folens et al., 2017
Melbourne (Australia)	108	0.1–1.0 (0.5)	12.9–723.0 (102.2)	11.6–49.4 (26)			5.9–7.5 (6.9)	92–637 (231)	Kandic et al., 2019
Christchurch (New Zealand)	50	0.33–10.7 (1.20)	22.6–2615 (137)	14.1–133 (22.7)	4.34–72.2 (12.1)	0.03–308 (0.29)			Ashrafzadeh et al., 2017
Marseille, Grand Nancy and Nantes (France)	104	0.1–8.9 (0.5)	19.5–566 (98.6)	21.3–136 (55.3)	0.2–65.2 (21.1)		6.2–8.3 (7.6)	17–103 (49)	Joimel et al., 2020
Marrakech (Morocco)	11	0.07–0.36 (0.20)	8.12–58.68 (23.12)	23.6–86.09 (42.83)	5.13–11.87 (7.71)		7.0–8.5 (7.9)	138–271 (220.5)	Laaouidi et al., 2020
**Regulation values**									
China		0.3	90	150	40	1.8	5.5–6.5		CEPA (2018)
EC		1.5	100	100					EC (2000)
WHO/FAO		3–6	250–500	100	20				WHO/FAO (2007)

Although the origin of PTEs in soils may be either lithogenic or anthropogenic, in most cases, high PTE concentrations are associated with human-induced activities^50,51^. The results from *I*_geo_ and *PI* calculations both indicated that the soils of Chengdu City were lesser enriched with Cr relative to the other elements (Fig. 2), suggesting that Cr in this study area originate from a natural source; this conclusion was consistent with a published result^22^. However, the results of Spearman correlation analysis, PCA, and CA indicated that Cd, Pb, and Hg exclusively (and As partially) had a common source, which may imply anthropogenic inputs, which would be consistent with previous reports^27,52^. In the present study, Cd and As clearly contributed the most to soil contamination in the investigated area compared with the other elements (Fig. 2). These results were similar to previous studies, which were conducted on urban vegetable garden soil of other regions. For example, the mean level of Cd in garden vegetable soils from Xiangyang City in northeast China was 2.13 times higher than the background value^52^. Moreover, the Cd concentration in urban garden soils in China was reported to be higher than the concentrations of other PTEs^53^. Antoniadis et al. found that the As concentration in garden vegetable soils in Cologne was 5.0–86.2 times higher than the background value^54^. It was generally acknowledged that there are many factors that contribute to Cd and As accumulation in urban vegetable soils, including irrigation with domestic and industrial wastewater, aerial deposition of industrial dust plums and automobile exhaust, application of pesticides and fertilizers to the soil, and other garden practices^54^. Typically, Cd is considered as a toxic element marker of agronomic practices, including the use of chemical fertilizers and livestock, which represent key sources of Cd introduction into soil^55^. According to a previous report^56^, inorganic As compounds, such as sodium arsenate, calcium arsenate, and many others were widely applied as herbicides and pesticides, thereby becoming important sources of As in urban vegetable soils.

### PTE contamination in vegetables

The varied in metal concentrations among the studied vegetables implied that the vegetable species had unique abilities and capacities to take up and accumulate the PTEs. The mean Cd, Pb, and As concentrations in leafy vegetables were higher than those in other vegetable categories. Among the leafy vegetables, the Cd concentrations in the Chinese cabbage and lettuce leaves were significantly higher than in other vegetables, and the Pb and As concentrations in Chinese cabbage and amaranths were the highest, with mean values of 0.051 and 0.067 mg/kg FW, respectively. Chaturvedi et al. reported that spinach plants (a leafy vegetable) could accumulate high amounts of Cd, Pb, and As in the edible parts, and no visible signs of toxicity were observed, which was consistent with the BAF results of the present study (Table S2)^37^.

The highest level of Hg in vegetables was found in rootstocks, particularly in the Houttuynia cordatas. This was consistent with a report from Wang et al. who detected higher Hg concentrations in Houttuynia cordatas compared to five other vegetables, and the highest Hg concentration was 14.5 times greater than the permissible level (0.01 mg/kg FW)^35^. Several studies have reported Hg concentrations in rootstock vegetables, along with some potential accumulation mechanisms. Carrasco-Gil et al. found that phytochelatins contributed to the retention of Hg in the roots owing to their ability to sequester Hg and facilitate Hg transport from cytosol to vacuole, thereby immobilizing Hg in the roots^37^. However, uptake of Hg is species-dependent^57^. Indeed, the concentration of Hg in Houttuynia cordatas was 9.02 times higher than that in carrots despite the fact that these were both categorized as rootstock vegetables. The Cr concentration in tomatoes was higher than in all other vegetables.

The relationships between the PTE concentrations in vegetables and their growing soil characteristics are presented in Table 4. A significant positive correlation for As (*r* = 0.387, *p* < 0.01) was observed, indicating that the soil As concentration could likely somewhat predict the amount of As in the edible parts of the vegetables. Negative correlations were observed for Cr, Cd, and Hg, indicating that the soil-to-plant transfer factors decreased with increasing concentrations of these elements in vegetables^58^, especially Cr (*r* = –0.398, *p* < 0.01). However, the total PTE concentration in soil may not be the only crucial factor controlling the metal bioavailability and vegetable uptake; these aspects also depend on other geochemical variables^59^. It is important to note from Table 4 that significant negative correlations were found between pH and Cd, Pb, As, and Hg, while SOM was negatively correlated with Cd, Pb, and As. Kirkham et al. and Tsadilas et al. pointed out that the Cd content in plants decreased as the soil pH increased^60,61^. Additionally, the uptake of Pb by the three vegetable types increased in acidic soil environments^62^.

**Table 4:** Spearman correlation matrix for soil characteristics and PTEs in vegetables (*n* = 113).

	pH	SOM	S(Cd)	S(Pb)	S(Cr)	S(As)	S(Hg)
V(Cd)	− 0.710**	− 0.342**	− 0.057	− 0.028	0.146	0.362**	− 0.120
V(Pb)	− 0.423**	− 0.414**	0.090	0.019	0.260**	0.420**	− 0.219*
V(Cr)	0.147	0.045	− 0.191*	0.015	− 0.398**	− 0.063	− 0.044
V(As)	− 0.402**	− 0.371**	− 0.045	− 0.039	0.017	0.387**	− 0.130
V(Hg)	− 0.441**	− 0.153	− 0.124	− 0.034	0.046	0.348**	− 0.073

*Significant correlation at 0.05 level.

**Significant correlation at 0.01 level.

### Health risks for local people

Evaluating the human risk associated with contaminated vegetable consumption also requires relating the problems of PTE pollution with the vegetable consumption trends. The EDIs of the studied metals were determined according to the mean concentration of each metal in each vegetable species, and considering the respective consumption rate for each species^63^. Some recent reports have confirmed that contaminated vegetables may be significant contributors to the total dietary PTE intake, particularly in terms of Cd, As, and Pb^25,55,64^. In the present study, it may seem comforting that the PTEs concentrations in vegetables were often below the maximum permissible limits. Nevertheless, there was a significant increase in health risks associated with exposure to these toxic elements, especially for Cr and As (Fig. 4). This may be attributed to the relatively high EDIs of Cr and As (Table S3). The total intake of Cd, Pb, Cr, As and Hg via vegetables consumption were found to be 0.20, 0.25, 0.31, 0.30 and 0.01 mg/kg/day for adult whereas 0.37, 0.46, 0.58, 0.57 and 0.02 mg/kg/day for children, respectively (Table S3). The total EDIs of Cd, Pb, Cr and As were 4.75, 1.14, 1.5 and 2.5 times higher than the maximum tolerable daily intake (MTDI) for adult whereas 7.82, 2.19, 2.85 and 4.66 times higher for children indicated that these metals could pose potential health risk via vegetables consumption in the study area.

The hazard index results suggested an overall non-carcinogenic health effect of the toxic elements on human. It is important to note that HI values for lettuce leaves, amaranths, Chinese cabbage and Houttuynia cordatas surpassed the threshold level (HI > 1) for adults and children (Fig. 4a,b). Several other reports found HI values greater than one due to vegetable consumption^18,19,64^. In terms of individual metals, As contributed significantly to the non-carcinogenic health hazard, followed by Cd and Cr. The results highlighted that people in the study area could encounter severe non-carcinogenic health risks following consumption of the studied vegetables.

The calculated carcinogenic health risk associated with Cr and As ingestion from consuming the studied vegetables indicated that tomatoes, lettuce leaves and amaranths induced serious cancer risks exceeding the threshold value in adults and children (Fig. 4c,d). In terms of individual metals, Cr exhibited a higher cancer risk than As. The total carcinogenic risk of the studied vegetables was higher than the USEPA standard (10^–4^), thus confirming the cancer risk to adults and children in the study area^65,66^. Several study was reported similar cancer risk values based on vegetable consumption^18,19,64^. However, it is difficult to understand the true potential human health risks based on total PTE concentrations without considering the bioaccessible forms of the metals^18^. Alarmingly, the present study revealed that consumption of contaminated urban garden vegetables would definitely pose carcinogenic health risks on its consumers through the combined effects of the toxic elements present after being taken up from the soil.

## Conclusions

Urban vegetable gardening is common, but exposure to PTEs originating from industrial activities or irrigation with polluted water tainted by domestic sewage or heavy traffic requires urgent attention. The mean concentration of metals in soil was reported in the decreasing order of Cr > Pb > As > Cd > Hg. Most of samples exceeded the background values for PTEs. Vegetables were also contaminated with metals. Higher Cd concentration was found in lettuce leaves (0.04 mg/kg FW) and Pb in Chinese cabbage (0.05 mg/kg FW), Cr in tomatoes (0.07 mg/kg FW), As in amaranths (0.07 mg/kg FW), respectively. The pollution load index values indicated that the soils in urban gardens were polluted with PTEs, often beyond the threshold limits. Estimated daily intake of metals due to vegetables consumption reveled that total EDIs of Cd, Pb, Cr and As were 4.75, 1.14, 1.5 and 2.5 times higher than the maximum tolerable daily intake (MTDI) for adult whereas 7.82, 2.19, 2.85 and 4.66 times higher for children. The health risk assessment also confirmed that exposure to Cd and As presented the greatest non-carcinogenic risk (> 1), while Cr exposure was associated with the highest cancer risk (> E04). Furthermore, the risk of exposure was even higher for children compared with adults. Therefore, the results of this study indicate that more regulative and preventative measures should be adopted by community health care services to combat further accumulation of these metals in the urban vegetable gardens of southwest China.

## Supplementary Information

Supplementary Information.

## Data Availability

The datasets used and/or analyzed during the current study are available from the corresponding author on reasonable request.
