# Supplementary material for: Assessment of the pollution levels of potential toxic elements in urban vegetable gardens in southwest China
Source: Sci Rep. 2021 Nov 24;11:22824. doi: 10.1038/s41598-021-02069-6 (PMC8613288; doi:10.1038/s41598-021-02069-6)
Supplement: Supplementary file 1 — Supplementary Information. [file 41598_2021_2069_MOESM1_ESM.docx]

**Assessment of the pollution levels of potential toxic elements in urban vegetable gardens in southwest China**

Jianing Gao,^1,2^ Dan Zhang,^1,^* Ram Proshad,^1,2^ Ernest Uwiringiyimana,^1,2^ and Zifa Wang^3^

^1^ Key Laboratory of Mountain Surface Processes and Ecological Regulation, Institute of Mountain Hazards and Environment, Chinese Academy of Sciences, Chengdu, China

^2^ University of Chinese Academy of Sciences, Beijing, China

^3^ Institute of Atmospheric Physics, Chinese Academy of Sciences, Beijing, China

*** Corresponding author: Dan Zhang**

#9, Block 4, Renminnanlu Road, Chengdu, Sichuan Province, 610041, the People's Republic of China; Email: [daniezhang@imde.ac.cn](mailto:daniezhang@imde.ac.cn)


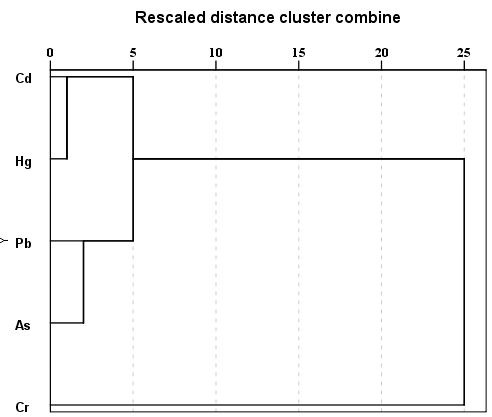


**Fig. S1.** Dendrogram of hierarchical cluster analysis of PTEs in vegetable soils of Chengdu City (*n* = 113).

**Fig. S2**. The internal standard curve for ICP-MS (Internal standard is the rhodium standard solution, Rh, 5% HCl).

**Table S1**. Principal component analysis matrix for PTE concentrations in vegetables soils in Chengdu City (*n* = 113).

| Heavy metals | Component | |
| --- | --- | --- |
|  | PC1 | PC2 |
| Cd | 0.726 | 0.443 |
| Pb | 0.675 | 0.359 |
| Cr | 0.747 | -0.104 |
| As | 0.479 | -0.589 |
| Hg | -0.265 | 0.769 |
| Eigenvalue | 1.842 | 1.273 |
| % of Variance | 36.83 | 25.47 |
| Cumulative % | 36.833 | 62.3 |

**Table S2**. Concentrations of PTEs in four categories of vegetables and BAF values.

| Vegetable categories | Concentration (mg/kg on FW basis) | | | | |  | Concentration (mg/kg on DW basis) | | | | |  | BAF | | | | |
| --- | --- | --- | --- | --- | --- | --- | --- | --- | --- | --- | --- | --- | --- | --- | --- | --- | --- |
|  | Cd | Pb | Cr | As | Hg |  | Cd | Pb | Cr | As | Hg |  | Cd | Pb | Cr | As | Hg |
| Leafy | 0.035±0.032^**^ | 0.041±0.013^*^ | 0.031±0.020 | 0.052±0.022 | 0.0014±0.001^*^ |  | 0.14±0.13^**^ | 0.16±0.05^*^ | 0.12±0.08 | 0.21±0.09^**^ | 0.006±0.002^*^ |  | 0.801±1.229^**^ | 0.005±0.003^*^ | 0.001±0.001 | 0.007±0.003^*^ | 0.136±0.122^*^ |
| Rootstock | 0.013±0.008 | 0.025±0.008 | 0.018±0.004 | 0.029±0.017 | 0.0016±0.001^*^ |  | 0.05±0.03 | 0.01±0.03 | 0.09±0.03 | 0.12±0.07 | 0.006±0.005^*^ |  | 0.369±0.261 | 0.003±0.001 | 0.001±0.001 | 0.004±0.002 | 0.088±0.07 |
| Legume | 0.012±0.003 | 0.014±0.010 | 0.026±0.009 | 0.014±0.010 | 0.0003±0.00 |  | 0.05±0.01 | 0.06±0.04 | 0.10±0.04 | 0.06±0.04 | 0.001±0.001 |  | 0.288±0.165 | 0.002±0.001 | 0.001±0.001 | 0.003±0.002 | 0.021±0.011 |
| Solanaceous | 0.007±0.001 | 0.006±0.017 | 0.065±0.011^**^ | 0.003±0.001^**^ | 0.0007±0.00 |  | 0.03±0.00 | 0.03±0.01 | 0.26±0.05^**^ | 0.01±0.00 | 0.003±0.001 |  | 0.160±0.071 | 0.001±0.001 | 0.004±0.001^**^ | 0.001±0.001 | 0.043±0.015 |

* and ** represent significant difference at the level of 0.05 and 0.001, respectively.

**Table S3**. The average estimated daily intakes (EDIs) of PTEs (FW) in different vegetables (mg/kg/day).

|  | Cd | |  | Pb | |  | Cr | |  | As | |  | Hg | |
| --- | --- | --- | --- | --- | --- | --- | --- | --- | --- | --- | --- | --- | --- | --- |
|  | adults | children |  | adults | children |  | adults | children |  | adults | children |  | adults | children |
| Amaranths | 0.026 | 0.049 |  | 0.047 | 0.087 |  | 0.054 | 0.101 |  | 0.081 | 0.151 |  | 0.0009 | 0.0017 |
| Chinese cabbage | 0.047 | 0.087 |  | 0.061 | 0.115 |  | 0.012 | 0.022 |  | 0.047 | 0.087 |  | 0.0021 | 0.0040 |
| Lettuce leaves | 0.053 | 0.098 |  | 0.036 | 0.066 |  | 0.057 | 0.106 |  | 0.068 | 0.127 |  | 0.0018 | 0.0033 |
| Carrots | 0.008 | 0.015 |  | 0.029 | 0.053 |  | 0.023 | 0.044 |  | 0.023 | 0.044 |  | 0.0004 | 0.0007 |
| Houttuynia cordatas | 0.024 | 0.045 |  | 0.031 | 0.058 |  | 0.021 | 0.038 |  | 0.048 | 0.089 |  | 0.0034 | 0.0064 |
| Cowpeas | 0.012 | 0.023 |  | 0.009 | 0.016 |  | 0.031 | 0.058 |  | 0.026 | 0.049 |  | 0.0003 | 0.0006 |
| Pea pods | 0.018 | 0.033 |  | 0.027 | 0.051 |  | 0.032 | 0.060 |  | 0.007 | 0.013 |  | 0.0005 | 0.0009 |
| Tomatoes | 0.008 | 0.015 |  | 0.008 | 0.014 |  | 0.078 | 0.146 |  | 0.003 | 0.006 |  | 0.0008 | 0.0015 |
| All vegetables | 0.196 | 0.365 |  | 0.248 | 0.46 |  | 0.308 | 0.575 |  | 0.303 | 0.566 |  | 0.0102 | 0.0191 |
| Maximum tolerable daily intake (MTDI) | 0.046^a^ | |  | 0.21^a^ | |  | 0.2^b^ | |  | 0.126^a^ | |  | 0.04^c^ | |

^a^JECFA (JECFA 2003); ^b^RDA (RDA 1989); ^c^Arora et al., 2008

**References**

JECFA 2003. Summary and conclusions of the 61st meeting of the Joint FAO/WHO. Expert Committee on Food Additives (JECFA). JECFA/61/SC. Geneva, Switzerland.

RDA. 1989. National Research Council (US) Subcommittee on the Tenth Edition of the Recommended Dietary Allowances. Washington (DC): National Academies Press (US).

Arora M, Kiran B, Rani S, Rani A, Kaur B, Mittal N. Heavy metal accumulation in vegetables irrigated with water from different sources, Food Chem. 2008; 111:811–815. https://doi.org/10.1016/j.foodchem.2008.04.049

**Table S4**. The condition parameters for ICP-MS instrument.

| Description | STD Mode | KED Mode | DRC Mode | Setting Time | Minmum Value | Maxmum Value |
| --- | --- | --- | --- | --- | --- | --- |
| Nebulizer Gas Flow | 0.91 | 0.91 | 0.94 | 10 | 0 | 1.5 |
| Auxiliary Gas Flow | 1.2 | 1.2 | 1.2 | 10 | 0.6 | 2 |
| Plasma Gas Flow | 18 | 18 | 18 | 10 | 10 | 20 |
| ICP RF Power | 1148 | 1148 | 1148 | 15 | 500 | 1600 |
| Analog Stage Voltage | -1950 | -1950 | -1950 | 2 | -3000 | 0 |
| Pulse Stage Voltage | 1300 | 1300 | 1300 | 2 | 0 | 2500 |
| Discriminator Threshold | 12 | 12 | 12 | 0 | 0 | 1000 |
| Deflector Voltage | -9 | -9 | -9 | 0 | -100 | 20 |
| Quadrupole Rod Offset | 0 | -12 | -7.5 | 1 | -26 | 26 |
| Cell Entrance Voltage | -6 | -6 | -5 | 1 | -60 | 20 |
| Cell Exit Voltage | -6 | -33 | -5 | 1 | -60 | 20 |
| Cell Rod Offset | -8 | -15 | -2 | 1 | -40 | 10 |
| Axial Fied Voltage |  | 475 | 250 |  |  |  |
| RPa |  | 0 | 0 |  |  |  |
| RPq |  | 0.25 | 0.45 |  |  |  |
| Cell Gas A |  | 2 | 0 |  |  |  |

**Table S5**. The information of quality assurance and quality control.

| No. | Heavy metals | Standard curve equation | *R*^2^ | Spectral intensity range (cps) | Relative standard deviation range | Recovery rates of the standard substances range (%) |
| --- | --- | --- | --- | --- | --- | --- |
| 1 | Cd | *y*=2 363.7*x* + 322.5 | 0.9996 | 4 473 - 235 920 | -0.0037 - 0.042 | 97.30 - 103.82 |
| 2 | Pb | *y*=9 717.2*x* + 606.9 | 0.9992 | 4 109 - 962 436.8 | -0.0001 - 0.014 | 95.47 - 104.28 |
| 3 | Cr | *y*=12 754*x* + 9735.4 | 0.9999 | 19 144 - 1 280 242 | 0.0002 - 0.005 | 96.00 - 103.42 |
| 4 | As | *y*=580.2*x* + 23.8 | 0.9999 | 586.6 - 5 834.8 | -0.042 - 0.053 | 98.02 - 102.42 |
| 8 | Hg | *Y* 4 359.4*x*-13.5 | 0.9999 | 421.6 - 4 361.8 | -0.0036 - 0.0059 | 96.55 - 102.77 |

**Table S6**. The information about certified reference materials analysis.

| Heavy meatals | Description | CAS | Concentration | Standard solution medium |
| --- | --- | --- | --- | --- |
| Cd | GSB 04-1721-2004 | 203006-1 | 1 000 μg/mL | c(HNO3)=1.0 mol/L |
| Pb | GSB 04-1742-2004 | 19C045-1 | 1 000 μg/mL | c(HNO3)=1.0 mol/L |
| Cr | GSB 04-1723-2004 | 216022-7 | 1 000 μg/mL | H2O |
| As | GBW(E) 080989 | ZBR331 | 1 000 μg/mL | 5% H2SO4 |
| Hg |  | 7439-97-6 | 10 mg/L | 5% HNO3 |

Note: Cd, Pb, Cr, and As Hg were purchased from National Research Center of Reference Materials (China), and e were purchased from the Institute of geophysical and geochemical exploration (China).

**Table S7**. Statistical analysis of soil and vegetable raw data.

|  |  | Vegetable (mg/kg on FW basis) | | | | |  | Soil (mg/kg on DW basis) | | | | | | |
| --- | --- | --- | --- | --- | --- | --- | --- | --- | --- | --- | --- | --- | --- | --- |
|  |  | Cd | Pb | Cr | As | Hg |  | pH | SOM | Cd | Pb | Cr | As | Hg |
| Sample number |  | 113 | 113 | 113 | 113 | 113 |  | 113 | 113 | 113 | 113 | 113 | 113 | 113 |
| Normal Parameters^a,b^ | Mean | 0.022 | 0.028 | 0.031 | 0.033 | 0.001 |  | 6.551 | 55.632 | 0.218 | 35.290 | 90.912 | 29.574 | 0.061 |
|  | Std. Deviation | 0.025 | 0.017 | 0.020 | 0.025 | 0.001 |  | 0.865 | 26.991 | 0.125 | 14.329 | 19.546 | 13.119 | 0.021 |
| Most Extreme Differences | Absolute | 0.242 | 0.068 | 0.134 | 0.121 | 0.175 |  | 0.133 | 0.077 | 0.197 | 0.165 | 0.135 | 0.147 | 0.053 |
|  | Positive | 0.242 | 0.066 | 0.134 | 0.121 | 0.175 |  | 0.063 | 0.077 | 0.197 | 0.165 | 0.135 | 0.147 | 0.044 |
|  | Negative | -0.217 | -0.068 | -0.095 | -0.105 | -0.149 |  | -0.133 | -0.060 | -0.173 | -0.129 | -0.062 | -0.078 | -0.053 |
| Kolmogorov-Smirnov Z |  | 2.569 | 0.724 | 1.422 | 1.288 | 1.859 |  | 1.409 | 0.814 | 2.097 | 1.751 | 1.431 | 1.563 | 0.558 |
| Asymp. Sig. (2-tailed) |  | 0.000 | 0.671 | 0.035 | 0.073 | 0.002 |  | 0.038 | 0.521 | 0.000 | 0.004 | 0.033 | 0.015 | 0.914 |

^a^ Test distribution is Normal.

^b^ Calculated from data.
